# Exercise Training-Induced PPARβ Increases PGC-1α Protein Stability and Improves Insulin-Induced Glucose Uptake in Rodent Muscles

**DOI:** 10.3390/nu12030652

**Published:** 2020-02-28

**Authors:** Ju-Sik Park, John O. Holloszy, Kijin Kim, Jin-Ho Koh

**Affiliations:** 1 Department of Taekwondo, College of Physical Education, Keimyung University, Daegu 42601, Korea; parkjs@kmu.ac.kr; 2 Division of Geriatrics and Nutritional Sciences, Department of Medicine, Washington University School of Medicine, St. Louis, MO 63110, USA; 3 Department of Physical Education, College of Physical Education, Keimyung University, Daegu 42601, Korea; 4 Department of Physiology, College of Medicine, Yeungnam University, Daegu 42415, Korea

**Keywords:** exercise training, protein stability, PPARβ, PGC-1α, GLUT4, Mitochondria, skeletal muscle

## Abstract

This study aimed to investigate the long-term effects of training intervention and resting on protein expression and stability of peroxisome proliferator-activated receptor β/δ (PPARβ), peroxisome proliferator-activated receptor gamma coactivator 1-α (PGC1α), glucose transporter type 4 (GLUT4), and mitochondrial proteins, and determine whether glucose homeostasis can be regulated through stable expression of these proteins after training. Rats swam daily for 3, 6, 9, 14, or 28 days, and then allowed to rest for 5 days post-training. Protein and mRNA levels were measured in the skeletal muscles of these rats. PPARβ was overexpressed and knocked down in myotubes in the skeletal muscle to investigate the effects of swimming training on various signaling cascades of PGC-1α transcription, insulin signaling, and glucose uptake. Exercise training (Ext) upregulated PPARβ, PGC-1α, GLUT4, and mitochondrial enzymes, including NADH-ubiquinone oxidoreductase (NUO), cytochrome c oxidase subunit I (COX1), citrate synthase (CS), and cytochrome c (Cyto C) in a time-dependent manner and promoted the protein stability of PPARβ, PGC-1α, GLUT4, NUO, CS, and Cyto C, such that they were significantly upregulated 5 days after training cessation. PPARβ overexpression increased the PGC-1α protein levels post-translation and improved insulin-induced signaling responsiveness and glucose uptake. The present results indicate that Ext promotes the protein stability of key mitochondria enzymes GLUT4, PGC-1α, and PPARβ even after Ext cessation.

## 1. Introduction

Fatty acid and glucose are the primary energy sources used during skeletal muscle contraction. During exercise, these sources must be supplied to myocytes to regulate muscle contraction in accordance with the intensity and duration of exercise, and the capacity of glucose uptake and fatty acid oxidation in the muscle adaptively increases in response to a bout of exercise. Glucose uptake in response to muscle contractions is mediated by glucose transporter type 4 (GLUT4) translocation to the cell membrane [1,2], and an adaptive increase in GLUT4 induces an increase in maximally stimulated muscle glucose transport activity [3,4]. Exercise training (Ext) reportedly upregulates GLUT4 [5] and mitochondrial proteins, such as NADH-ubiquinone oxidoreductase (NUO), cytochrome c oxidase subunit 4 (COX4) [6], cytochrome C (Cyto C), and citrate synthase (CS) [7]. Further, the energy-generating capacity of the mitochondria with substrates, such as glucose and fatty acids, influence glucose uptake and fatty acid oxidation levels, and the expression levels and half-life of mitochondrial enzymes are reportedly differently regulated [6,7], indicating that protein stability is different during and after Ext. A bout of exercise upregulates GLUT4 [3], albeit not to peak expression levels. Further, mitochondria are not completely adapted following a single bout of exercise [7]. Therefore, GLUT4 expression and mitochondrial biogenesis could be gradually enhanced to supply energy to maintain muscle contractions in response to a bout of exercise during Ext. Therefore, we considered investigating time-dependent mechanisms underlying the changes in the expression of these proteins upon Ext.

One of the cardinal benefits of exercise training (Ext) is the regulation of whole-body glucose homeostasis, which is differently regulated via distinct mechanisms between before exercise and recovery from exercise. During an exercise bout, the activated muscle displays increased glucose uptake in an insulin-independent manner [8]. Furthermore, after an exercise bout, skeletal muscle insulin sensitivity and responsiveness are enhanced even at rest [9]. Increased GLUT4 transporters and mitochondria proteins are generally the key factors associated with increased glucose uptake and fatty acid metabolism in skeletal muscles; however, it remains unclear how much of this increase in protein levels is attributed to increased expression or potentially enhanced protein stability after Ext termination. Furthermore, it remains unclear how long the beneficial effects of Ext on glucose homeostasis during recovery from exercise last. 

We previously reported that peroxisome proliferator-activated receptor β/δ (PPARβ) is an upstream factor regulating GLUT4 expression [5] and mitochondrial biogenesis [6]. PPARβ expression is induced through exercise training, and peroxisome proliferator-activated receptor gamma coactivator 1-alpha (PGC-1α) is a transcriptional co-activator, serving as a key factor for mitochondrial biogenesis and is increased by both acute exercise [10] and exercise training [6]. However, it remains unclear how these proteins are expressed in a time-dependent manner upon Ext. Time-course data are important to determine whether the expression and interaction of these proteins are regulated through known pathways during Ext. Therefore, this study aimed to investigate the expression of PPARβ, PGC-1α, GLUT4, and mitochondrial enzymes over 28 days of Ext in rats and after 5 days of resting (detraining). We also determined whether PGC-1α, GLUT4, and the mitochondrial enzymes are regulated by PPARβ using mice. An understanding of how GLUT4 and mitochondrial enzymes are regulated during Ext would help identify potential therapeutic targets to improve systemic glucose management.

## 2. Materials and Methods 

### 2.1. Animals and Diets 

This study was approved (20140254) by the Animal Studies Committee of Washington University School of Medicine (St. Louis, MO, USA). Wistar male rats weighing ~100 g were purchased from Charles River (Wilmington MA, USA), housed (two to three rats per cage), and provided ad libitum access to Purina chow and water. Male C57BL/6J mice weighing ~20 g were purchased from Jackson Laboratory (Bar Harbor, ME, USA). Mice were housed (two or three per cage) and then housed in individual cages when animals required a stable environment after undergoing electroporation, and provided ad libitum access to Purina chow and water. 

### 2.2. Cell Lines

Cell culture experiments using a mouse skeletal muscle myocytes (C_2_C_12_) were carried out as previously described [6]. Briefly, the C_2_C_12_ mouse muscle cell line was obtained from ATCC (Manassas, VA, USA) and cultured in 5% CO_2_ at 37 °C in Dulbecco’s Modified Eagle Medium (DMEM) (Cat; D5796, Sigma-Aldrich, MO, USA) supplemented with 10% fetal bovine serum (FBS, Sigma-Aldrich, MO, USA), penicillin (100 U/mL), and streptomycin (100 µg/mL) (Thermo Fisher Scientific, MA, USA). Myoblasts differentiated into myotubes upon changing the medium to that containing 2% horse serum, penicillin (100 U/mL), and streptomycin (100 µg/mL) (Thermo Fisher Scientific, MA, USA). Plasmid DNA was transfected using Lipofectamine 2000 (Thermo Fisher Scientific, MA, USA).

### 2.3. Insulin Responsiveness

To determine the maximum insulin responsiveness, myotubes were incubated with serum and glucose-free media for 2 h, followed by 1 h incubation in serum-free medium containing 100 nM insulin. Myotubes were then harvested and assessed for AKT serine/threonine kinase (AKT) activation as a marker of the insulin response.

### 2.4. Exercise Programs

Ext involved an initial acclimatizing phase for swimming for 10 min per day for 3 days prior to the exercise regime. In the Ext, mice were made to swim in steel barrels filled to a depth of ~60 cm of water maintained at ~35 °C. For acute Ext, mice swam four times, each lasting 30 min, with a 5-min rest period between bouts. After the first bout, a weight equal to 1.5% of the body weight of the mouse was tied to the base of its tail and mice swam with the weight attached for the remaining 3 bouts. For exercise training for 28 and 5 days of de-training, six rats at each time point swam in two 3-h daily sessions, separated by a 45-min rest period. Rodents were anesthetized via intraperitoneal injection of 60 mg/kg body weight sodium pentobarbital and muscles were dissected out 24 h after exercise for protein analyses and immediately following exercise for mRNA quantification after cessation of exercise training. 

### 2.5. DNA Constructs and Muscle Electroporation

The PPARβ plasmids were constructed as previously described [6]. Briefly, PPARβ cDNA was amplified via PCR and inserted into the pcDNA 3.1 vector (Thermo Fisher Scientific) to generate the overexpression vector, and shPPARβ and scrambled shRNA (Scr) were purchased from Origene (Rockvile, MD, USA) and cloned into the same vector backbone. The efficiency of these vectors on PPARβ expression was previously validated [5,6]. The PPARβ overexpression plasmid, EV, shPPARβ plasmid, or the Scr plasmid was transfected into the mouse tibialis anterior (TA) muscle, using an electric pulse-mediated gene transfer method [6]. Mice were anesthetized via isoflurane inhalation. Thereafter, 50 µg of plasmid DNA containing either the empty vector or the PPARβ vector in a 50-µL injection solution was intramuscularly administered to a TA muscle, using a 27-gauge needle at a rate of 20 µL/min. Thereafter, an electric field was applied to the muscle, using an S88 stimulator (Grass instrument, West Warwick, RI, USA) [6]. Animals were anesthetized via intraperitoneal injection of sodium pentobarbital and muscles were dissected out, frozen in liquid nitrogen, and stored at 80 °C until analysis. 

### 2.6. Western Blot Analysis

Protein expression was analyzed via standard Western blot analysis, as previously described [5]. Briefly, frozen muscles were powdered and then homogenized in a 15:1 (vol./wt.) ratio of ice-cold buffer supplemented with a protease inhibitor cocktail tablet (Santa Cruz Biotechnology, Santa Cruz, CA, USA). Protein concentration was determined using Lowry’s method [11]. Antibodies used herein are as follows: anti-GLUT4 (Abcam, Cambridge, MA, USA), anti-PPARβ, anti-NADH ubiquinone oxidoreductase (NUO), and anti-cytochrome c oxidase subunit I (COXI) (Thermo Fisher Scientific, MA, USA), anti-PGC-1α (Millipore, Billerica, MA, USA), anti-β-actin (cat. no. A5441; Sigma Aldrich, MO, USA), anti-citrate synthase (CS; Alpha Diagnostics San Antonio, TX, USA), anti-cytochrome c (Cyto c; BD Biosciences, San Jose, CA, USA), anti-p38MAPK, anti-phospho-p38MAPK, anti-activating transcription factor 2 (ATF2), and anti-phospho-ATF2 (Cell Signaling Technologies, Danvers, MA, USA); and secondary antibodies including donkey anti-mouse (cat. no. 715-035-150), donkey anti-rabbit (cat. no. 711-035-152), and anti-streptavidin (cat. no. 016-030-084) antibodies (Jackson Immunoresearch Laboratories, West Grove, PA, USA). Antibody-bound protein was detected using the Clarity Western ECL Substrate (Bio-Rad, cat. no. 170-5060, Hercules, CA, USA). Signals were visualized using a C-DiGit blot scanner (Li-COR Bioscience, cat. no. 3600-00, Lincoln, NE, USA). 

### 2.7. Gene Expression Analysis

Total RNA was isolated from muscle using an RNeasy mini kit (Qiagen, Venlo, The Netherlands) in accordance with the manufacturer’s instructions, and cDNA synthesis was performed using the SuperScript IV First-Strand Synthesis System for RT-PCR (Thermo Fisher Scientific). Quantitative RT-PCR was performed using a thermocycler (Applied Biosystems, CA, USA). Samples were amplified in duplicate with the following PGC-1α primers: F; AAT GCA GCG GTC TTA GCA CT, R; GTG TGA GGA GGG TCA TCG TT. β-actin primers: F; TAC TGC CCT GGC TCC TAG CA, R; TGG ACA GTG AGG CCA GGA TAG. PGC-1α expression levels were normalized relative to those of β-actin.

### 2.8. Glucose Uptake

shPPARβ, PPARβ, or the control vector (Scramble or green fluorescence protein (GFP)) were transfected into myoblasts, and serum-free medium was changed after 4 days of differentiation. After 24 h, this medium was replaced with that containing 250 µM insulin without serum or glucose, and the myotubes were incubated for 1 h in the cell incubator. Thereafter, the medium was removed and 50 μL of 0.1 mM 2-Deoxy-D-glucose (2DG) in PBS was added, and myotubes were incubated for an additional 30 min at 25 °C. Glucose uptake was measured using Glucose Uptake-Glo Assay (Promega, MI, USA) in accordance with the manufacturer’s instructions. 

### 2.9. Immunofluorescence Analysis for GLUT4 Localization

Immunofluorescence staining for GLUT4 was performed as previously described [5]. Briefly, mouse muscles were dissected 30 min after an intraperitoneal (IP) injection of insulin, and then placed in OCT compound (Tissue-Tek, VWR, IL, USA). Embedded muscles were cooled with liquid nitrogen and stored at −80 °C. The muscles were cut into 10-µm-thick cryosections with a Cryostat (Thermo Electronic, PA, USA). GLUT4 localization was evaluated using multicolor immunofluorescence analysis [12]. The sections were co-incubated with primary anti-GLUT4 and anti-dystrophin antibodies for 2 h at room temperature (RT). The secondary antibodies (488-conjugated goat anti-rabbit IgG for GLUT4, and 594-conjugated goat anti-mouse IgG2b for dystrophin; Thermo Fisher Scientific, MA, USA) were applied to the sections for 30 min at RT. The fluorophores were visualized using a Nikon Eclipse TE2000-U (Nikon, Melville, NY, USA) and X-cite 120 PC (Excelitas, Waltham, MA, USA). The images were captured using the MetaMorph software (Molecular Devices, CA, USA). 

### 2.10. Statistical Analysis

Statistical analyses were conducted using SigmaPlot v.12 software (Systat, San Jose, CA, USA). Normally distributed data were analyzed utilizing standard parametric statistics, including Student’s t test or one-way ANOVA, followed by Tukey’s test for post hoc analysis. Data are expressed as means ± SE, and statistical significance was accepted when *p* < 0.05. 

## 3. Results

### 3.1. Exercise Training Upregulates GLUT4, PPARβ, and PGC-1a Protein Expression and those Half-Lives in Resting State

**Figure 1:**
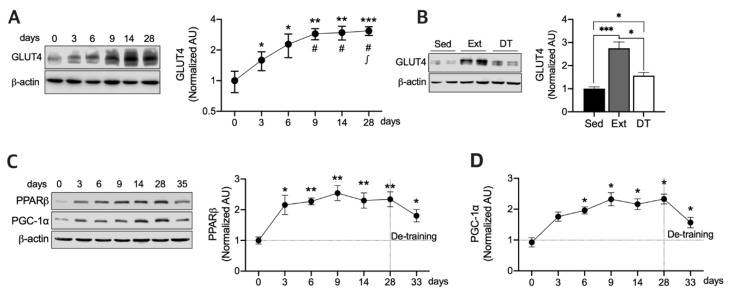
**Exercise training** (Ext) gradually upregulates glucose transporter type 4 (GLUT4), peroxisome proliferator-activated receptor β/δ (PPARβ), and peroxisome proliferator-activated receptor gamma coactivator 1-alpha (PGC-1α) and increases their stability. (**A**) GLUT4 protein expression was analyzed in a time-dependent manner at 0, 3, 6, 9, 14, and 28 days of exercise training (*n* = 6 muscles of rats per group). Values are means ± SE. * *p* < 0.05, ** *p* < 0.01, *** *p* < 0.001 versus day 0. ^#^ *p* < 0.01 versus day 3. ^∫^ *p* < 0.05 versus day 6. (**B**) GLUT4 protein expression was analyzed at 28 days of exercise training and after 5 days of de-training (DT) (*n* = 6 muscles of rats per group). Sed–Sedentary. Values are means ± SE. * *p* < 0.05, *** *p* < 0.001. (**C,D**). PPARβ and PGC-1α expression were analyzed in a time-dependent manner at 0, 3, 6, 9, 14, 28 of exercise training and 5 days de-training (*n* = 6 muscles of rats per group). Values are means ± SE. * *p* < 0.05, ** *p* < 0.01 versus day 0. All muscles were isolated 24 h after each training session. Significance was determined using one-way ANOVA.

We evaluated changes in protein and mRNA levels in rats that swam daily for 3, 6, 9, 14, or 28 days and in rats that rested for 5 days after training for 28 days to investigate the effect of de-training. We assessed GLUT4 protein expression in the rats at each time point after training cessation and after 5 days of de-training. Consequently, GLUT4 was gradually upregulated until day 9 of training (*p* < 0.05) and then stabilized during the rest of the training period (*p* < 0.01) (Figure 1A). Furthermore, GLUT4 expression levels remained significantly higher (*p* < 0.05) after resting for 5 days in comparison with the sedentary rats (Figure 1B), although GLUT4 expression levels were significantly lower (*p* < 0.05) than those after 28 days of training. PPARβ plays a key role in maintaining PGC-1α protein stability [6] and exercise-induced GLUT4 expression [5]. Moreover, PGC-1α mediates the exercise-induced GLUT4 upregulation and mitochondrial biogenesis [9]. Therefore, we assessed PPARβ and PGC-1α expression levels in a time-dependent manner to determine whether their expression levels display the same patterns as for GLUT4 during Ext (Figure 1A,B). As shown in Figure 1C,D, PPARβ was significantly upregulated (*p* < 0.5) upon Ext from day three, whereas PGC-1α expression levels were significantly higher (*p* < 0.05) than those in sedentary rats after 6 days of training (Figure 1D). Together, these data indicate that the expression of PGC-1α and PPARβ is differentially induced by Ext. Although acute exercise reportedly upregulates PGC-1α by 2.5-fold [10], herein, PGC-1α expression levels were slightly but not significantly higher than those in sedentary rats subjected to 3 days of Ext (Figure 1D). Both PPARβ and PGC-1α expression levels were still significantly higher (*p* < 0.05) than those in the sedentary rats after 5 days of termination of Ext (Figure 1C,D). Together, these results indicate that GLUT4 and PGC-1α protein expression induced by Ext is stabilized after approximately 5 days of exercise. PPARβ potentially influences the stability of PGC-1α during Ext because PPARβ is required to maintain normal levels of PGC-1α following Ext [6]. 

### 3.2. PPARβ Is Required to Maintain Normal Glucose Uptake and Maximal Insulin Sensitivity in Myotubes 

**Figure 2:**
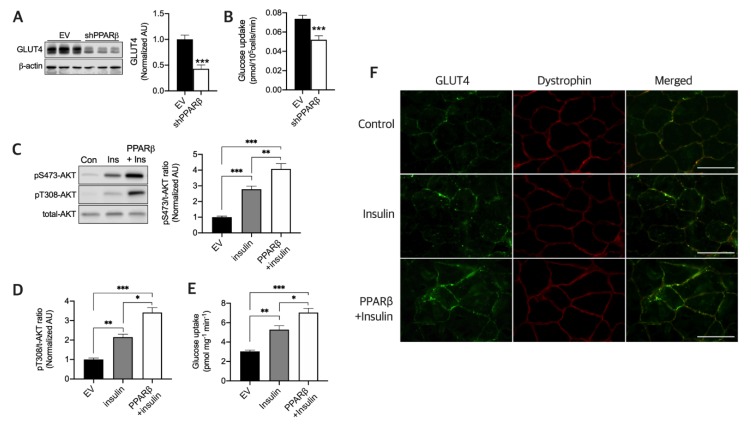
PPARβ is required to maintain normal glucose uptake and maximize insulin sensitivity. (**A**) Silencing of PPARβ (shPPARβ) downregulated GLUT4 (*n* = 6 myotubes per group). (**B**) Silencing of PPARβ (shPPARβ) decreased glucose uptake (*n* = 9 myotubes per group). (**C–E**) PPARβ synergistically increases insulin-induced AKT activation and glucose uptake (*n* = 6 myotubes per group). Values are means ± SE. * *p* < 0.05, ** *p* < 0.01, *** *p* < 0.001. (**F**) PPARβ enhances insulin-induced GLUT4 localization to the plasma membrane. Scale bar, 100 µm. Significance was determined using one-way ANOVA or Student’s *t*-test.

A previous study reported that PPARβ plays an important, but not essential, role in GLUT4 expression induced by Ext in the skeletal muscle [5]. We hence investigated how Ext-induced PPARβ regulates glucose uptake in the skeletal muscle in the resting state. To investigate the effect of PPARβ silencing on GLUT4 expression and glucose uptake in myotubes, shPPARβ was transfected in myotubes, and we found that GLUT4 expression (Figure 2A) and glucose uptake (Figure 2B) were both significantly decreased (*p* < 0.001) in comparison with the Scr plasmid. PPARβ overexpression in skeletal muscles reportedly upregulates GLUT4 [5]. Furthermore, we evaluated AKT signaling induced by insulin with or without PPARβ and found that PPARβ synergistically increases insulin-induced serine (*p* < 0.01) and threonine AKT phosphorylation (*p* < 0.05) (Figure 2C,D) and glucose uptake in myotubes (*p* < 0.05) (Figure 2E), along with GLUT4 translocation in the skeletal muscle of mice (Figure 2F). These data indicate that PPARβ is essential and that it maximized insulin sensitivity in the resting state.

### 3.3. A Post-Translational Cascade Induced by PPARβ Influences PGC-1α Protein Levels During and After Ext 

**Figure 3:**
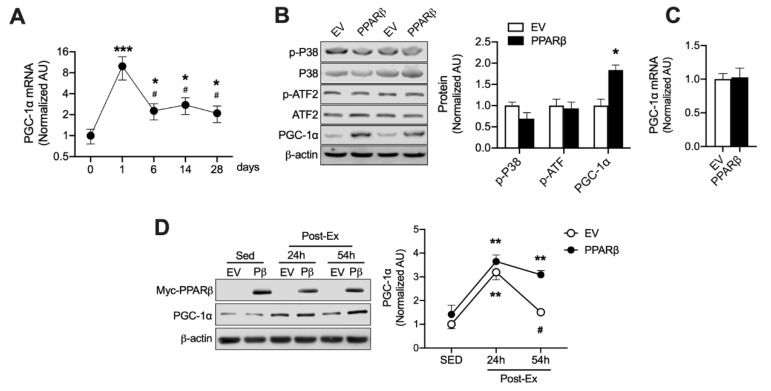
PPARβ regulates PGC-1α protein levels in a post-transcriptional manner. (**A**) PGC-1α mRNA is rapidly increased by a bout of exercise, and then downregulated at 6, 14, and 28 days of exercise training (*n* = 6 muscles of mice per each day), Values are means ± SE. * *p* < 0.05, *** *p* < 0.01 versus day 0. # *p* < 0.05 versus 1 day of exercise training. Muscles were isolated immediately after Ext. (**B**) p38, ATF2 and PGC-1α expression levels were determined in PPARβ-overexpressing skeletal muscles. Muscles were isolated 21 days after PPARβ overexpression through electroporation (*n* = 4 muscle of mice per group). Values are means ± SE. * *p* < 0.05 versus EV. (**C**) PPARβ overexpression does not increase PGC-1α mRNA (*n* = 4 muscle of mice per group). Values are means ± SE. (**D**) PPARβ overexpression increases PGC-1α protein stability in a post-transcriptional manner, empty vector (EV) or myc-PPARβ (Pβ) was overexpressed in the tibialis anterior, and muscles were isolated at 24 and 54 h after acute exercise (*n* = 6 muscles of mice per group). Values are means ± SE. ** *p* < 0.01 versus sedentary (Sed). # *p* < 0.01 verses PPARβ 54 h after acute exercise. Significance was determined using one-way ANOVA or Student’s *t*-test.

PGC-1α is rapidly upregulated by acute exercise and then rapidly reverts to baseline levels upon lack of stimulation [13,14,15] because PGC-1α is rapidly degraded in the nucleus via the ubiquitin-proteasome system (UPS) [16]. We previously reported that PPARβ binds to PGC-1α and blocks PGC-1α ubiquitination [6]. We speculated that Ext-induced PPARβ upregulation can improve PGC-1α protein stability and thus gradually increase protein levels during Ext. We found that Ext upregulated PGC-1α mRNA (approximately 8-fold) (*p* < 0.001) following a bout of exercise, and that PGC-1α mRNA levels after 6, 14, and 28 days of Ext were significantly decreased (*p* < 0.05) by approximately 6-fold rather than after acute exercise, although the mRNA levels were significantly higher (*p* < 0.05) than those in the sedentary state (Figure 3A). This finding is consistent with a report wherein high-intensity exercise upregulated PGC-1α mRNA by over 10-fold after the initial exercise session and then progressively decreased to a 4-fold increase over day 0 after the seventh session [17]. Despite these mRNA levels, the PGC-1α protein levels gradually increased during chronic exercise and displayed an approximately 2-fold increase by day 6 (Figure 1D), indicating that PGC-1α protein expression is regulated by a post-translational mechanism during Ext. PPARβ overexpression does not upregulate PGC-1α in skeletal muscles [18,19]. This finding was confirmed (Figure 3C); however, PPARβ overexpression in myotubes increased PGC-1α mRNA levels [20]. The mechanism underlying these differences between myotubes and skeletal muscles may be explained on the basis of the differences in p-38 MAPK signaling. Therefore, we determined whether PPARβ influences p38 and ATF pathways, since these are upstream factors for PGC-1α transcription [21]. Interestingly, overexpression of PPARβ in skeletal muscles slightly but not significantly reduced the phosphorylation of p-38 MAPK, and no change was observed in ATF2 phosphorylation (Figure 3B). However, PPARβ overexpression resulted in an increase in PGC-1α protein expression (*p* < 0.05) in the skeletal muscle (Figure 3B). Furthermore, we investigated the effect of PPARβ on PGC-1α protein stability using a different method from that reported previously [6]. Mice were exercised using a swimming program, resulting in a ~2-fold increase in PGC-1α protein expression (*p* < 0.01) regardless of PPARβ overexpression in their skeletal muscle, indicating that PPARβ did not influence the synthesis of PGC-1α protein upon acute exercise. Fifty-four hours after the exercise, PGC-1α levels reverted to baseline in muscles electroporated with the empty vector (EV) but were maintained at this high level in muscles, wherein PPARβ was overexpressed, indicating that PPARβ inhibited PGC-1α degradation after acute exercise (Figure 3D). These data indicate that PPARβ upregulation induced by Ext can prevent PGC-1α degradation and can increase PGC-1α protein levels in a post-transcriptional manner (Figure 3D), concurrent with our previous report with a different exercise intensity and type [22]. 

### 3.4. Mitochondrial Enzymes Are Differently Regulated by Ext

**Figure 4:**
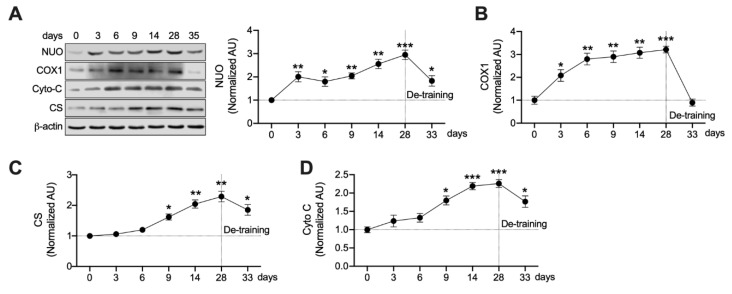
Differences in the normalization rates of mitochondrial proteins. (**A**,**B**) NUO and COX 1 are rapidly upregulated by Ext; however, only NUO is stable after 5 days of de-training (*n* = 6 muscles of rats per each day). Values are means ± SE. * *p* < 0.05, ** *p* < 0.01, *** *p* < 0.001 versus day 0. (**C**,**D**) CS and Cyto C are gradually upregulated by Ext and stabilize after 5 days of de-training (*n* = 6 muscles of rats per each day). Values are means ± SE. * *p* < 0.05, ** *p* < 0.01, *** *p* < 0.001 versus day 0. Significance was determined using one-way ANOVA.

Ext significantly increased PPARβ and PGC-1α expression in a time-dependent manner (Figure 1C,D). Since both PPARβ and PGC-1α increase mitochondrial biogenesis in skeletal muscles [6,9], we analyzed the expression of key mitochondrial enzymes in a time-dependent manner. We observed that the expression levels of each enzyme were normalized to those after Ext but with different rates of normalization (Figure 4). As shown in Figure 4, NUO (*p* < 0.01) and COX1 (*p* < 0.05) were rapidly and significantly upregulated after 3 days of exercise training and were stable during Ext (Figure 4A,B), whereas the expression levels of CS and Cyto C were significantly increased (*p* < 0.05) by Ext after 9 days of training, indicating a gradual increase during Ext (Figure 4C,D). Furthermore, NUO (Figure 4A), CS (Figure 4C), and Cyto C (Figure 4D) protein levels were still significantly higher (*p* < 0.05) than those in the sedentary state after 5 days of resting.

## 4. Discussion

GLUT4 and mitochondrial enzymes are the primary factors determining the rate of glucose and fatty acid utilization in skeletal muscles. A cardinal adaptation to endurance Ext is an increase in the biogenesis of mitochondrial enzymes and GLUT4 in skeletal muscles, increasing the potential for glucose and fatty acid metabolism during Ext and better disposal capacity of circulating glucose and fatty acid in the resting state. The molecular mechanism and protein expression in a time-dependent manner responsible for the adaptation to Ext is not fully understood. PPARβ and PGC-1α are key factors cooperating during Ext for regulating gene expression associated with glucose and fatty acid metabolism. The primary findings of this study are that Ext-induced PPARβ upregulation increased PGC-1α protein stability in skeletal muscles and maximized glucose uptake via enhanced GLUT4 expression and insulin responsiveness in myotubes. 

GLUT4 [5] and numerous mitochondrial enzymes [6] are reportedly upregulated via Ext-induced PPARβ upregulation. Herein, PPARβ was essential for maintaining normal levels of GLUT4 and glucose uptake in myotubes (Figure 2), concurrent with our pervious report that PPARβ is important for maintaining normal physiological GLUT4 protein expression following Ext [5], although PPARβ is not essential to induce GLUT4 expression by Ext [5]. Furthermore, herein, Ext upregulated GLUT4 in a time-dependent manner, and this protein stabilized during training and even after a 5-day resting session. In this context, a previous study reported that glucose uptake tended to be higher in mice at 7 days after resistance training for 16 weeks than in sedentary mice [23]. However, we could not elucidate the mechanism underlying the stabilization of GLUT4 for 5 days without Ext; however, GLUT4 has a long half-life in muscle cells [24], and the N-glycan chain on GLUT4 contributes to quality control of newly synthesized GLUT4 [25]. Further, N-glycosylation probably increases protein stability [26]. GLUT4 upregulation by PPARβ is essential for glucose uptake into myocytes (Figure 2A,B). Skeletal muscle is the primary tissue regulating postprandial glucose uptake [9], and PPARβ can maximize insulin-induced glucose uptake via enhanced AKT signaling (Figure 2C–E) and GLUT4 translocation (Figure 2F). Thus, PPARβ is essential for the improved capacity of insulin responsiveness and glucose uptake by Ext.

The acute increase in PGC-1α translocation to the nucleus that occurs in response to muscle contraction allows PGC-1α to bind to the specific nuclear receptor for energy metabolism-related gene expression. Since PGC-1α is a transcriptional co-activator [9], and is known to be a short-lived protein owing to its rapid degradation via the UPS [16], PGC-1α is expected to cooperatively bind to certain factors to protect itself against the UPS and to maintain gene expression in response to muscle contraction. We previously reported that PPARβ binds PGC-1α, blocking the ubiquitin-binding site on PGC-1α, and thus protecting it against ubiquitin binding and degradation. Finally, PPARβ overexpression increased PGC-1α levels [6]. Moreover, we found additional strong evidence that PPARβ overexpression in skeletal muscles increases protein stability and enhances PGC-1α protein expression (Figure 3D) without PGC-1α transcription (Figure 3C), indicating that PPARβ increases PGC-1α protein levels via a post-translational mechanism. As shown in Figure 3A, PGC-1α mRNA drastically increased following a bout of exercise; however, the mRNA level was only approximately 2-fold that in the sedentary state after 6, 14, and 28 days of training, indicating that exercise intensity is not enough to further stimulate the expression of PGC-1α mRNA. In rats undergoing endurance training at the same exercise intensity for 28 days, an adaptive increase in the skeletal muscles was almost accomplished with 6 days of training to perform at a particular exercise intensity. Furthermore, PPARβ upregulation caused by Ext limited p38 activation, which is upstream of PGC-1α transcription, as evident in Figure 3B. These results provide strong evidence that PPARβ upregulation induced by Ext protects the PGC-1α protein against degradation via the UPS, and PPARβ and PGC-1α cooperatively upregulate metabolism-related genes, such as GLUT4 and mitochondrial enzymes, since PPARβ is a nuclear receptor and PGC-1α is a transcriptional co-activator.

A previous study reported that PGC-1α is required to fully mature mitochondria, despite PPARβ upregulating certain mitochondrial enzymes without PGC-1α [6]. Herein, Ext rapidly increased NUO and COX1 levels and gradually increased the normalization of CS and Cyto C (Figure 4). PPARβ was rapidly upregulated following Ext (Figure 1C); however, PGC-1α protein was stabilized by 6 days of training (Figure 1D). CS and Cyto C levels peaked upon Ext (Figure 4C,D). Furthermore, NUO, CS, and Cyto C levels were still higher than those in a sedentary state after 5 days of resting (Figure 4). We believe that these levels are associated with an increase in protein stability induced by Ext, concurrent with a previous report that CS and Cyto C have higher half-lives in response to a constant exercise stimulus [7]. Together, these findings suggest that PGC-1α and PPARβ cooperatively enhance mitochondrial adaptation based on exercise intensity, and the protein stability of GLUT4 and mitochondrial enzymes are enhanced for 5 days after Ext.

In conclusion, this study shows that Ext upregulates PPARβ with an increase in the training dose and this upregulation mediates PGC-1α protein stability during Ext and 5 days after Ext cessation in a post-translational manner. Furthermore, PPARβ and PGC-1α cooperatively mediate GLUT4 expression and mitochondrial adaptation, and Ext upregulates GLUT4 and mitochondrial enzyme stability for 5 days after Ext termination via an unknown mechanism. The present results suggest that post-translational cooperative effects of PPARβ and PGC-1α in response to Ext mediate an adaptive increase in GLUT4 levels and mitochondrial biogenesis in skeletal muscles, thus contributing to glucose homeostasis via increased responsiveness to insulin and GLUT4 translocation.
